# THE BURDEN OF SPOUSAL CAREGIVERS IN YOUNG-ONSET DEMENTIA: INSIGHTS FROM A MIXED-METHODS STUDY

**DOI:** 10.1093/geroni/igae098.4340

**Published:** 2024-12-31

**Authors:** Xiaoyan Cui, Junqiao Wang, Bei Wu, Ding Ding, Qianhua Zhao, Xueting Tang, Jing Wang

**Affiliations:** Fudan University, Shanghai, Shanghai, China (People’s Republic); Fudan University, Shanghai, Shanghai, China (People’s Republic); New York University, New York, New York, United States; Huashan Hospital, Fudan University, Shanghai, Shanghai, China (People’s Republic); Huashan Hospital, Fudan University, Shanghai, Shanghai, China (People’s Republic); Fudan University, Shanghai, Shanghai, China (People’s Republic); University of New Hampshire, Durham, New Hampshire, United States

## Abstract

Becoming a spousal caregiver for a person with young-onset dementia (YOD) is an unexpected event that deviates from the normal life course trajectory, creating unique and profound challenges. Understanding their burden, the primary sources of this burden, and their coping resources is essential for developing tailored support services. We employed a mixed-methods approach to gain a comprehensive understanding of the burden experienced by eleven spousal caregivers of persons with YOD living in the community. We used the Zarit Burden Interview to quantify caregiving burden and conducted semi-structured qualitative interviews to explore the caregiving experience in depth. We utilized a joint display method to integrate and present the converging findings from quantitative and qualitative data, offering a holistic perspective on the caregiving burden. All spousal caregivers in this study experienced varying degrees of burden, driven primarily by the dynamic care needs and dependency of their loved ones. The integrated data showed that they face significant challenges in maintaining their personal identity and social life, with many sacrificing career aspirations and enduring social isolation. Societal expectations compounded the burden, leading to self-imposed guilt and dissatisfaction. While caregiving reconfigures family dynamics, it often strengthens bonds with children. The lack of support resources and overwhelming responsibilities places immense psychological stress on them. Despite these challenges, caregivers remain committed to their roles. There is an urgent need to develop family-centered community support services and respite care to provide younger spousal caregivers with the time and space to manage their personal lives and alleviate their burden.

